# Nuclear Receptor Regulation of Aquaporin-2 in the Kidney

**DOI:** 10.3390/ijms17071105

**Published:** 2016-07-11

**Authors:** Xiao-Yan Zhang, Bing Wang, You-Fei Guan

**Affiliations:** 1 Advanced Institute for Medical Sciences, Dalian Medical University, Dalian 116044, China; wangbing_1999_1981@163.com (B.W.); guanyf@dmu.edu.cn (Y.-F.G.); 2 Department of Physiology, AstraZeneca–Shenzhen University Joint Institute of Nephrology, Shenzhen University Health Science Center, Shenzhen 518060, China; 3 Department of Physiology and Pathophysiology, School of Basic Medical Sciences, Peking University Health Science Center, Beijing 100191, China

**Keywords:** water channel, peroxisome proliferator-activated receptors (PPARs), liver X receptors (LXRs), farnesoid X receptor (FXR), estrogen receptor (ER)

## Abstract

Aquaporin-2 (AQP2) is a vasopressin-regulated water channel responsible for regulating water reabsorption through the apical plasma membrane of the principal cells of renal collecting ducts. It has been found that dysregulation and dysfunction of AQP2 cause many disorders related to water balance in people and animals, including polyuria and dilutional hyponatremia. Classically, AQP2 mRNA and protein expression and its membrane translocation are regulated by systemic vasopressin involving short-term regulation of AQP2 trafficking to and from the apical plasma membrane and long-term regulation of the total amount of the AQP2 protein in the cell. Recently, increasing evidence has demonstrated that collecting duct AQP2 expression and membrane translocation are also under the control of many other local factors, especially nuclear receptors. Here, we briefly review the progress of studies in this area and discuss the role of nuclear receptors in the regulation of water reabsorption via affecting AQP2 expression and function.

## 1. Introduction

Water is a basic necessity of life and is the main component of extracellular fluid. Normally, the body’s water content (50%–60% of body weight) and osmotic pressure of extracellular fluid (280–295 mOsm/kg) play a critical role in maintaining homeostasis, which is achieved through the regulation of water intake and loss. Daily water intake from drinking and food and endogenous production of water via the body’s metabolism provide approximate 1.5–3.0 L. Accordingly, water is excreted from the kidneys, lungs, skin and gastrointestinal tract to maintain water homeostasis of whole body. It is wildly accepted that urine produced from the kidney is the main way to mediate water output from the body. 

The kidney is a central organ in water homeostasis regulation through its ability to concentrate and dilute urine according to the hydration state of the body. In general, 180 L of water is filtered through the glomerulus of an adult human each day. Flowing through the tubules, more than 99% of the initial urine is reabsorbed, leaving about 1.5 L of terminal urine in daily urine output. The volume and osmotic pressure of terminal urine fluctuate with changes in the balance of water in the body. Even if water intake is not so low, or water intake is within normal range, urine could be more concentrated than blood plasma. Conversely, when water ingestion is large enough to dilute blood plasma, the urine that is produced is more dilute than blood plasma [1]. Renal dysfunction of urine concentration and dilution may result in isotonic urine, with a urine sodium concentration equal to that of plasma sodium regardless of the state of water balance.

Water reabsorption along the renal tubules is very important in maintaining body water homeostasis. Approximately 85% of the filtered water reabsorbed in the proximal tubules and the descending thin limbs of Henle, and the remaining 15% is reabsorbed in the connecting tubules and collecting ducts. Although a significant volume of water in glomerular filtrate is reabsorbed in the proximal tubules, the main renal tubules capable of regulating water reabsorption and urine concentration are the connecting tubule (CNT) and collecting ducts. In these renal tubules, the primary regulator of water reabsorption is the antidiuretic hormone (ADH) or arginine vasopressin (AVP), which acts on water channel aquaporins (AQPs), its main molecular targets. In particular, the AVP/AQP2 pathway in CNT and collecting ducts is believed to play critical roles in maintaining the body’s balance of water [2,3,4,5]. 

## 2. AQP Expression in the Kidney

AQPs belong to the family of water channel proteins. As transmembrane proteins, they are inserted into the cell membranes and act as the transporters for water and small solutes such as glycerol, gas and ions. To date, more than 13 AQPs in mammals have been identified [6,7,8,9], named AQP 0–12 respectively. AQPs are expressed in various tissues including the kidney, liver, brain, eye, lungs and salivary glands [10]. According to their transport capabilities, AQPs can be classified into 3 subtypes: (1) classical AQPs include AQP1, AQP2, AQP4, AQP5 and AQP8. They are water-selective channels and transport water only. In addition, AQP8 has been reported to act as an ammoniaporin and a peroxiporin [11]; (2) AQPs include AQP3, AQP7, AQP9, and AQP10. In addition to water, AQPs in this subtype are also permeated by small uncharged molecules such as glycerol and urea; (3) Unorthodox AQPs include AQP6, AQP11 and AQP12, whose functions are currently unknown [12,13]. A large amount of evidence demonstrates that AQP1, 2, 3, 4, 6, 7, 8, and 11 are expressed in the mammalian kidney [14], while only AQP1–4 have been proven to be important in renal water reabsorption [15]. Among them, AQP1 is expressed in the proximal tubules and the descending thin limbs of the long loop nephrons responsible for constitutive absorption of a large amount of water, while AQP2–4 are located in the principal cells of the connecting tubules and collecting ducts in the kidney, where they control water reabsorption and determine the final urine volume [16]. In particular, AQP2 is located in the apical and subapical vesicles of the luminal plasma membrane [17]. Patients with mutations of *AQP2* gene exhibit impaired urinary concentrating capacity and a polyuria phenotype [5,18]. In rodents, *AQP2* gene knockout mice die within the first few days of life and collecting duct-specific *AQP2* gene knockout mice exhibit severe polyuria [4,19]. Increasing evidence demonstrates that dysregulation of AQP2 disrupts whole body water balance resulting in many clinical conditions. It has been reported that many factors can influence the expression and translocation of AQP2 [20]. The regulation involves both short-term modulation that controls translocation of AQP2 proteins from intracellular vesicles to the apical membrane, and long-term regulation via the changes of *AQP2* gene transcription, protein stability and degradation [21].

## 3. The AVP-V2 Receptor System and Locally Produced Factors in Controlling AQP2 Expression

**Figure 1:**
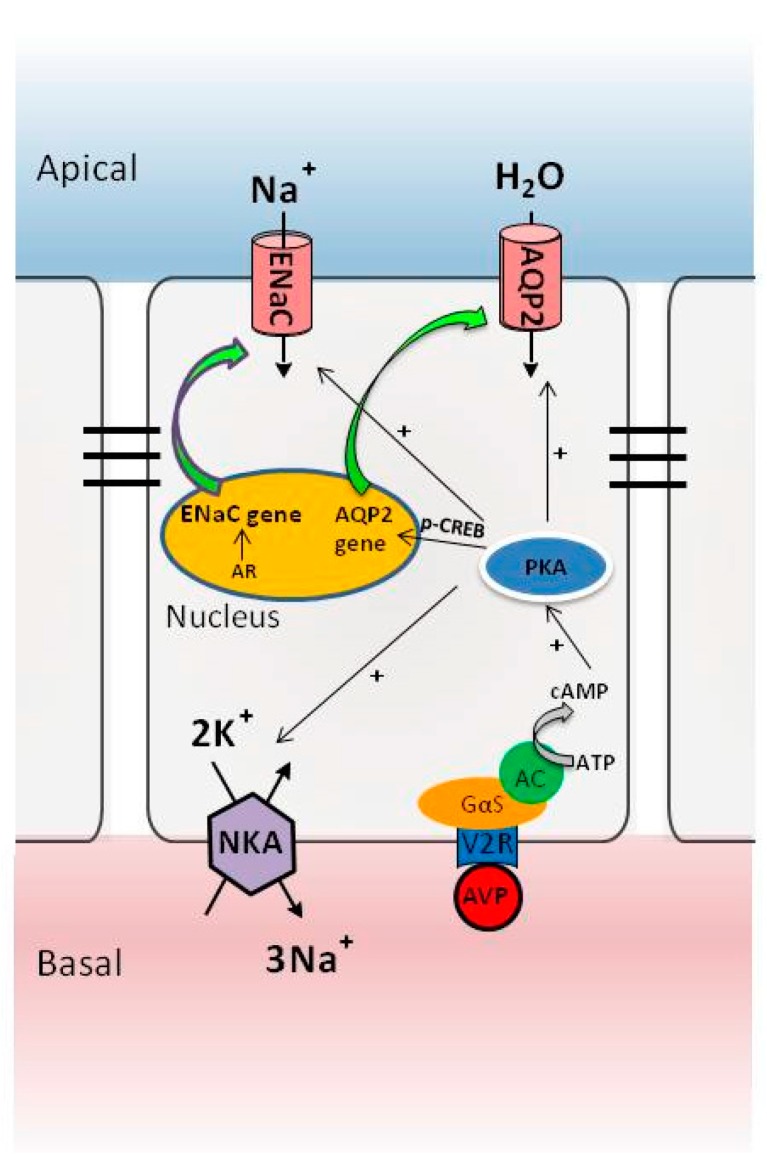
The arginine vasopressin (AVP)-V2 receptor system in controlling water and sodium reabsorption in renal collecting ducts. AR, aldosterone receptor; NKA, Na^+^-K^+^-ATPase; +, activation.

It has been well documented that AQP2 is primarily regulated by the AVP produced in the hypothalamus, stored in the posterior pituitary [22]. Increased plasma osmolality and decreased blood volume are believed to be two major factors inducing the release of AVP from the pituitary. AVP binds to its V2 receptor located in the basolateral membrane of the principal cells of the collecting ducts and triggers the classical cAMP-PKA pathway, which acutely increases water reabsorption via increasing AQP2 phosphorylation at serines 256, 264, and 269 and chronically upregulates *AQP2* gene transcription through the phosphorylated activation of cAMP-responsive element-binding protein (CREB) transcription factor [20]. Recently, increasing evidence have demonstrated that AVP also stimulates Na^+^ reabsorption by coordinated activation of ENaC and Na^+^-K^+^-ATPase at the post-translational level in an aldosterone-independent manner (Figure 1). Loss of function mutations in V2R causes a severe disease named nephrogenic diabetes insidipus, characterized by kidney inability to respond to the hormone vasopressin. In contrast, gain of function mutations is associated with the syndrome of inappropriate vasopressin secretion [23].

In addition to AVP, AQP2 is highly regulated in a complex manner. There are many other identified factors capable of regulating the translocation of AQP2 protein and expression of *AQP2* gene, such as extracellular osmotic pressure, insulin, nuclear factor κB (NFκB), renin-angiotensin-aldosterone system (RAAS), kinins, nitric oxide, adenosine, ATP and endothelins [20,24,25,26,27,28]. Prostaglandin E2 (PGE2) has also been found to be an important local lipid mediator in controlling AQP2 expression and function [29,30,31]. We recently reported that ablation of collecting duct PGE2 receptor EP4 resulted in a polyuria phenotype [21]. Moreover, recent studies underscored the importance of microenvironmental factors in the regulation of AQP2, such as fluid shear stress and extracellular pH, or RNA interference [32,33,34]. Therefore, collecting duct AQP2 expression and translocation are under the control of many systemic and local factors. 

## 4. Nuclear Receptor Regulation of AQP2 in Collecting Ducts

**Figure 2:**
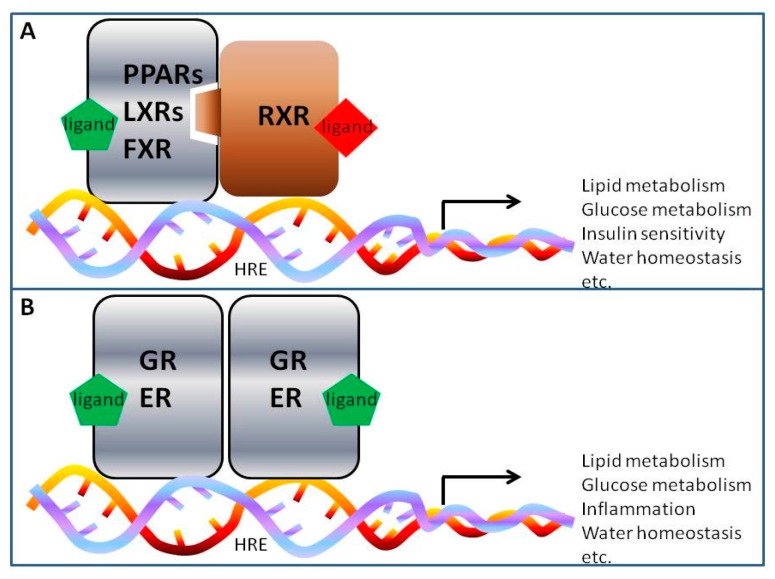
Schematic modes of nuclear receptor (NR) actions (**A**,**B**). HRE, hormone response element.

In the past decade, emerging evidence have demonstrated that many nuclear receptor transcription factors are actively involved in the regulation of water transport in renal collecting ducts and contribute to body water homeostasis. Nuclear receptors (NRs) are a group of ligand-activated nuclear transcription factors consisting of 48 members in humans [35]. They are functional in the nucleus and contain a few functional domains including the DNA-binding domain (DBD) and ligand-binding domain (LBD) [36]. Ligand-activated NRs can recognize and bind to specific DNA sequences known as the hormone response elements in the promoter regions of their target genes, thereby regulating target gene transcription and expression. One of the classic models for transcriptional regulation of NRs such as PPARs, liver X receptors LXRs and farnesoid X receptor FXR is that, upon binding of its ligand, NR forms a heterodimer with the retinoid-X-receptor (RXR), which then binds to the promoter region of its target genes resulting in an increase in its target gene transcription (Figure 2A). In addition, NR such as glucocorticoid receptor (GR), aldosterone receptor or mineralocorticoid receptor (MR) and ER can form a homodimer, which directly regulates the transcription of its target genes (Figure 2B).

NRs are involved in multiple physiological and pathological processes including cell growth, metabolic regulation, immune response and inflammation. Increasing evidence demonstrates that NRs may also take part in the regulation of water and sodium homeostasis. Peroxisome proliferator-activated receptor gamma (PPARγ), GR, mineralocorticoid receptor (MR) or aldosterone receptor (AR), FXR, liver X receptor beta (LXRβ), and estrogen receptor alpha (ERα) have been recently reported to be important in regulating water transport in renal collecting ducts [37,38,39,40,41]. In the present review, we will discuss the role of these NRs in regulating AQP2 expression and the underlying mechanisms.

### 4.1. Peroxisome Proliferator-Activated Receptor Gamma (PPARγ)

PPARs are members of the nuclear receptor superfamily and subdivided into 3 isoforms including PPARα, PPARβ/δ, and PPARγ. Previous studies have shown that dysfunction of PPARs is involved in the development of many metabolic diseases, such as insulin resistance, type 2 diabetes mellitus (T2DM) and hyperlipidemia. Synthetic agonists of PPARα such as fibrates have been used to decrease serum triglyceride levels in patients with hyperlipidemia. Activation of PPARδ has been experimentally shown to improve insulin resistance and reduce the urinary albumin excretion in a mouse model of diabetic nephropathy [42]. PPARγ agonist thiazolidinediones (TZDs) including rosiglitazone and pioglitazone are insulin sensitizers and have been clinically used to treat patients with T2DM. 

Although TZDs have established the ability to alleviate insulin resistance, their clinical use is associated with many serious side effects such as fluid retention, weight gain and increased risk of congestive heart failure. In the past decade, numerous studies have been carried out to define the mechanism by which PPARγ activation induces water retention and edema [39,43,44,45]. PPARγ is constitutively expressed in the kidney and abundantly located in the principal cells of inner medullary collecting ducts (IMCDs) [46], suggesting that this nuclear receptor may be involved in sodium and water transport in this renal segment. In addition to IMCDs, low but detectable levels of PPARγ are found in human proximal tubule cells [47]. 

In addition to diabetic patients, type 2 diabetic animals receiving TZD treatment also develop water retention. A study using a T2DM mouse model (db/db mice) revealed that treatment of db/db mice with rosiglitazone (RGZ) exhibited larger plasma volume than that of lean mice. RGZ-induced water retention activated natriuretic and diuretic mechanisms in lean mice, which was diminished in db/db mice. Imunoblotting analysis found that RGZ treatment significantly downregulated the expression of renal AQP2 in lean mice, which contributes to water excretion. However, downregulation of AQP2 by RGZ was markedly attenuated in the kidneys of db/db mice. Therefore, inappropriate regulation of AQP2 likely underlies much more severe water retention in db/db mice than in lean mice [48].

In 2005, Guan and Yang independently created a transgenic mouse line specifically lacking the *PPARγ* gene in the renal collecting ducts (CD-PPARγ knockout mice) and found that TZD-induced fluid retention was remarkably attenuated in these animals [39,43,44,49], suggesting that renal collecting duct PPARγ is very likely the molecular target of TZDs. To further characterize the molecular mechanisms involved in TZD-associated edema, Song et al. reported that activation of PPARγ by RGZ indeed induced rapid sodium and water retention in Sprague-Dawley rats, which exhibited reduced urine volume and decreased Na^+^ excretion. These changes were accompanied by increased expression of sodium transport proteins such as α-1 Na^+^-K^+^-ATPase, the sodium hydrogen exchanger (NHE3), the bumetanide-sensitive Na^+^-K^+^-2Cl^−^ cotransporter (NKCC2) and two members of AQPs (AQP2 and 3) in the kidney [45]. Using another potent PPARγ agonist GI262570, Chen L et al. found that PPARγ activation also increased AQP2 mRNA expression in rat renal medulla [50]. Consistently, Tiwari et al. also found that RGZ treatment reduced hematocrit levels and increased the expression of non-glycosylated AQP2 in the membrane-enriched fraction of the kidneys of Sprague-Dawley rats [51]. Together, these findings indicate that induction of *AQP2* gene transcription and increase in AQP2 membrane translocation in renal collecting ducts may play an important role in TZD-associated water retention [52].

In terms of the physiological role of PPARγ in the renal collecting duct, Procino et al. observed that RGZ promoted AQP2 trafficking to the apical plasma membrane in cultured MCD4 cells, probably mediated by increased Ca^2+^ influx by TRPV6. In an inducible PPARγ deficient mouse line generated by using the tamoxifen system, Zhou et al. reported that PPARγ knockout mice developed a polyuria phenotype and produced hypoosmotic urine with urinary AVP excretion unchanged, indicating a urine concentrating defect in these animals. DDAVP administration significantly increased urine osmolality in control floxed mice, but not in PPARγ knockout mice. Furthermore, the abundance of total and phosphorylated AQP2 in the kidney, together with AVP-induced cAMP accumulation were unaffected. This study demonstrates that PPARγ may take part in physiological regulation of water homeostasis independent of the AVP/cAMP/AQP2 pathway [44]. 

Taken together, although the precise mechanism by which PPARγ regulates renal water reabsorption remains only partially understood, increasing evidence suggests that increased expression of the *AQP2* gene and enhanced membrane targeting of the AQP2 protein play an important role in mediating PPARγ agonist-induced fluid retention, especially in T2DM. 

### 4.2. Glucocorticoid Receptor (GR)

GR belongs to the NR superfamily and is ubiquitously expressed in almost every cell type. GR controls the gene transcription of many target genes, and exerts diverse biological functions such as glucose, fat and protein metabolism, inflammation and immune response. All of the biologic actions are mediated by the binding of ligands including endogenous glucocorticoids (GCs) to the GR. GCs are steroid hormones, and are secreted by the adrenal cortex. They bind and activate the GR and exert both physiological and pathophysiological functions. Although GCs are traditionally considered as important anti-inflammatory molecules, increasing evidence suggests that GCs are also involved in water homeostasis regulation. 

It has been reported that in patients with adrenocortical insufficiency and animal models deficient for GCs, the nonosmotic release of AVP results in water retention, which can be corrected by glucosteroid replacement therapy [53,54,55]. In addition, in glucocorticoid deficient rats, AVP antagonist treatment increases water excretion after water loading [55]. These findings demonstrate that excessive AVP release is responsible for water retention in glucocorticoid deficiency. In 2000, Saito and colleagues reported that glucocorticoid deficiency increased renal medullary AQP2 expression in an AVP-dependent manner [56]. In addition to an increase in the total amount of AQP2 protein, phosphorylated AQP2 (pAQP2 at Ser256) protein abundance was also significantly upregulated in the inner medullas of glucocorticoid deficient rats compared to the control group. Even after water loading for 1 h, the glucocorticoid deficient rats showed increased trafficking of AQP2 to the apical plasma membrane, which was diminished by vasopressin V2 receptor antagonist [57]. 

Interestingly, excessive GCs (such as Cushing’s syndrome) can also induce water retention, which is mediated by the GR and results from increased expression of AQP2. Chen et al. demonstrated that dexamethasone, a synthetic glucocorticoid, increased expression of AQP2 in the inner medullas of adrenlectomized rats [58]. In vitro studies reported that dexamethasone upregulated the expression of AQP2 protein in rat inner medullary collecting duct (IMCD) suspension and reduced AQP2 protein degradation in cultured HEK293 cells [59]. Activation of GR also increased AQP2 cell membrane abundance, which can be blocked by RU486, an GR antagonist [59]. These studies provide direct evidence that glucocorticoid or GR may increase AQP2 expression and membrane translocation in the kidney. However, the molecular mechanism by which GR regulates AQP2 expression and water reabsorption in renal collecting ducts needs to be further clarified. 

### 4.3. Mineralocorticoid Receptor (MR)

As a member of the NR family, MR is also known as nuclear receptor subfamily 3, group C, member 2 (NR3C2) and can be activated by mineralocorticoids. Aldosterone is the main mineralocorticoid hormone which binds to the MR. Therefore, MR is also known as aldosterone receptor (AR) [60]. Once bound to its ligand, MR translocates to the nucleus, where it upregulates gene transcription of its target genes. Accumulating studies reveal that MR and its ligand aldosterone are the main modulators of sodium and water reabsorption in the kidney. 

To date, studies on the effect of aldosterone or MR on water reabsorption and AQP2 expression have shown contradictory results. Jonassen et al. reported that in normal rats, canrenoate (a chronic MR blocker) significantly increased the daily urine flow rate by 44% and decreased urine osmolality by 27%, with a significant decrease in AQP2 expression levels [61]. However, in rats with lithium-induced nephrogenic diabetes insipidus (Li-NDI), activation of MR markedly increased urine volume, whereas the blockade of MR with spironolactone decreased urine production. Compared with rats treated with lithium only, aldosterone reduced AQP2 expression in the apical plasma membrane in the connecting tubule (CNT) and initial cortical collecting ducts (iCCD). In contrast, treatment with spironolactone significantly increased apical AQP2 expression in iCCD [62]. In vasopressin-deficient BB rats, similar changes were observed [62]. Another study in mineralocorticoid deficiency rats showed a significant increase in the expression of AQP2 and AQP3 proteins in the inner medulla, but increased urine output. The explanation for increased urine output is that, besides water reabsortion by AQP2, decreased Na^+^-K^+^-2Cl^−^ co-transporter and Na^+^-K^+^-ATPase in renal outer medulla also contributed to urine concentration and dilution [63]. In addition, in a study of rats with aldosterone deficiency, renal AQP3 expression level was significantly decreased, whereas AQP2 level was unchanged [64]. This was accompanied by the reduction of basolateral infoldings of the cortical collecting duct principal cells. This finding may indicate that modulation of basolateral membrane area of the collecting duct principal cells could be an important component of aldosterone action in the kidney [64,65]. Previous studies also demonstrated that treatment of aldosterone resulted in dual effects on AQP2 expression in a time-dependent manner in mpkCCDC14 cells. Aldosterone reduced AQP2 mRNA and protein levels when administrated with AVP for short time (≤24 h) [25]. However, aldosterone incubation for 48 h increased AQP2 protein expression by increasing AQP2 mRNA translation [25]. Both events were mediated by the MR occupancy because these effects were induced by physiological concentrations of aldosterone (10^−9^ M) and were abolished by the MR antagonist canrenoate [66,67].

In addition, several studies on 11β-hydroxysteriod dehydrogenase type 2 (11β-HSD2) suggest a complex role of MR in water reabsorption. Aldosterone and cortisol have equal affinities to MR in vitro. The selective activation of MR by aldosterone in distal nephrons is based on the activity of 11-dehydrocorticosterone, which converts cortisol (an MR ligand) to cortisone. Humans with congenital loss of 11β-HSD2 develop an apparent excess of mineralocorticoids, hypertension, hypokalemic alkalosis and a suppressed renin-angiotensin-aldosterone system. Evans et al. reported that 11β-HSD2^−/−^ mice have a severe and progressive polyuric and polydipsic phenotype, with decreased AQP2 and AQP3 expression, which results from overactivation of MR [68].

### 4.4. Farnesoid X Receptor (FXR)

FXR is a nuclear receptor responsible for regulating bile acid, cholesterol, fatty acid, and glucose homeostasis. Previous studies have shown that FXR is mainly expressed in the liver and small intestine, where it plays an important role in bile acid and lipid metabolism. Recently, we have found that FXR is also highly expressed in the kidney and ubiquitously distributed in renal tubules, where it plays an important role in renal water reabsorption [69]. Activation of FXR dramatically lowered urine volume and increased urine osmolality, while FXR knockout mice exhibited a polyuria phenotype as a result of an impaired ability to concentrate urine. Further studies have shown that FXR endogenous ligand chenodeoxycholic acid (CDCA) treatment significantly up-regulated renal AQP2 expression in C57BL/6 mice, whereas FXR knockout mice exhibited markedly reduced AQP2 expression at both the mRNA and protein levels. In addition, luciferase reporter and Chip assays revealed that human and mouse *AQP2* gene promoter region has a putative FXR binding site (FXRE), suggesting that *AQP2* gene is a direct target gene of FXR. 

### 4.5. Liver X Receptors (LXRs)

The subfamily of nuclear receptor LXR has two members, i.e., LXRα and LXRβ. They are important regulators of lipid homeostasis and highly expressed in tissues with active lipid metabolism. Increasing evidence has suggested that LXRs may also be involved in regulating in urine concentration [70]. Administration of TO901317 (a non-selective LXR agonist) resulted in a urine concentration defect as reflected by polyuria and polydipsia in C57/Bl6 mice. This effect was associated with reduced (pro)renin receptor (PRR) and AQP2 expression in the kidney, which was reversed by the administration of PRR agonist [70]. These findings suggest that activation of LXRs may reduce AQP2 expression in renal collecting ducts. A recent study by Gabbi et al. showed that LXRβ, but not LXRα, plays an important role in controlling water balance. LXRβ^−/−^ mice exhibited classical symptom of diabetes insipidus, including polyuria and polydipsia due to decreased AVP expression in the magnocellular neurons, and GW3965 (another LXR agonist) treatment of wild-type mice increased urine osmolality [40]. Immunohistochemical study revealed that AQP1 expression was significantly reduced in the kidneys of LXRβ^−/−^ mice. This finding suggests that increased urine output in LXRβ^−/−^ mice may not only be due to defective expression of hypothalamus AVP, but also as a result of reduced renal AQP1 expression. However, it remains unclear whether AQP2 expression was reduced in LXRβ^−/−^ mice. The role of LXRα and LXRβ in regulating renal AQP2 expression also requires further investigation. 

### 4.6. Estrogen Receptor Alpha (ERα)

As a sex hormone, estradiol has been reported to be involved in the regulation of water and salt homeostasis. Water retention is common during pregnancy, and upregulation of AQP2 contributes to the water retention in pregnancy through a V2 receptor-mediated effect [71]. In ovariectomized (OVX) female rats, urine osmolality was increased with renal AQP2 phosphorylation significantly upregulated. Replacement of estradiol increased urine output, decreased urinary osmolality and reduced the expression and phosphorylation at Ser256 of AQP2 levels. In addition, native mouse collecting ducts and the mouse cortical collecting duct (mpkCCD) cell line express both ERα and ERβ. Estradiol treatment of mpkCCD cells reduced AQP2 at both the mRNA and protein levels in the absence of deamino-8-d-AVP (dDAVP) and significantly moderated the dDAVP-induced increase in AQP2 at the protein level only [41]. Further study revealed that mice lacking ERα displayed significant increases in AQP2 protein compared with wild-type controls, indicating the role of ERα in mediating the inhibitory effect of estradiol on AQP2 expression [41].

## 5. Perspective and Future Direction

**Figure 3:**
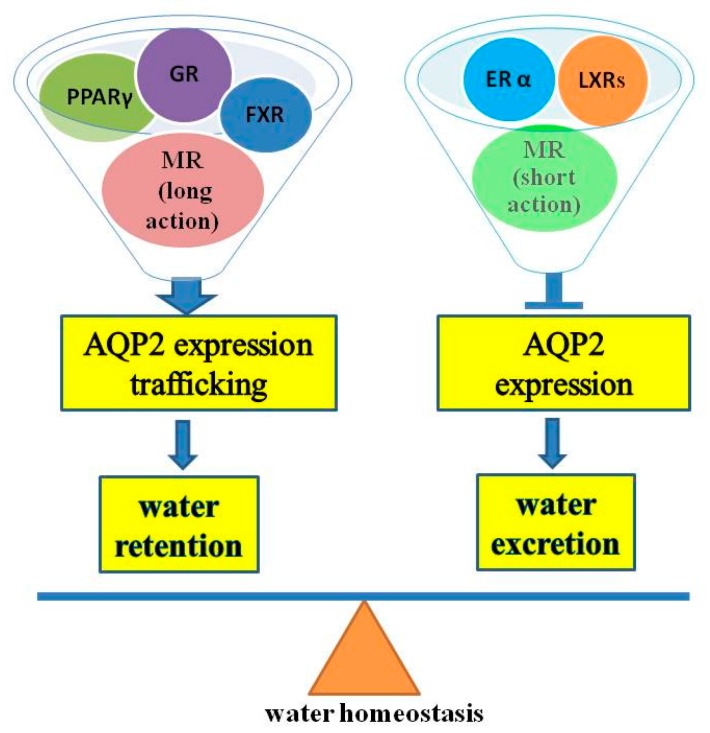
NRs in controlling *AQP2* gene expression and function.

As discussed above, AQP2 is the water channel predominantly expressed in the principal cells of renal collecting ducts and responsible for water reabsorption in this renal segment. AQP2 expression and membrane plasma trafficking are mainly regulated by systemic vasopressin. Recently, a large body of evidence demonstrates that nuclear receptors especially PPARγ, LXRβ, FXR, GR, MR and ERα play an important role in regulating AQP2 abundance and membrane translocation (Figure 3). Dysregulated nuclear receptors may therefore contribute to the development of disorders of water balance including body fluid retention and diabetes insipidus. Very likely, many other nuclear receptors may also be involved in the regulation of *AQP2* gene expression and protein modification in renal collecting ducts. It is also currently unclear how these nuclear receptors act in concert with the AVP/V2 receptor signaling pathway in maintaining bodily water homeostasis via fine-tuning collecting duct AQP2 protein expression and function. Defining the types of NRs involved in regulating AQP2 function and the mechanisms by which NRs control water reabsorption in renal collecting ducts is therefore essential for our understanding of many clinical disorders related to water imbalance.
